# Granzyme B mediates impaired healing of pressure injuries in aged skin

**DOI:** 10.1038/s41514-021-00059-6

**Published:** 2021-03-05

**Authors:** Christopher T. Turner, Juliana Bolsoni, Matthew R. Zeglinski, Hongyan Zhao, Tatjana Ponomarev, Katlyn Richardson, Sho Hiroyasu, Erin Schmid, Anthony Papp, David J. Granville

**Affiliations:** 1 International Collaboration on Repair Discoveries (ICORD) Centre, Vancouver Coastal Health Research Institute, University of British Columbia, Vancouver, BC Canada; 2 Department of Pathology and Laboratory Medicine, University of British Columbia, Vancouver, BC Canada; 3 Centre for Heart Lung Innovation, St. Paul’s Hospital, University of British Columbia, Vancouver, BC Canada; 4 Blusson Spinal Cord Wound Clinic, Vancouver Coastal Health, Vancouver, BC Canada; 5 Department of Surgery, University of British Columbia, Vancouver, BC Canada; 6 British Columbia Professional Firefighters’ Burn and Wound Healing Group, Vancouver, BC Canada

**Keywords:** Biochemistry, Geriatrics, Inflammation

## Abstract

Pressure injuries (PIs), also known as bedsores or pressure ulcers, are a major cause of death and morbidity in the elderly. The serine protease, Granzyme B (GzmB), contributes to skin aging and impaired wound healing. Aging is a major risk factor for PIs; thus, the role of GzmB in PI pathogenesis was investigated. GzmB levels in human PI tissue and wound fluids were markedly elevated. A causative role for GzmB was assessed in GzmB knockout (GzmB−/−) and wild-type (WT) mice using a murine model of PI. An apolipoprotein E knockout (ApoE−/−) model of aging and vascular dysfunction was also utilized to assess GzmB in a relevant age-related model better resembling tissue perfusion in the elderly. PI severity displayed no difference between young GzmB−/− and WT mice. However, in aged mice, PI severity was reduced in mice lacking GzmB. Mechanistically, GzmB increased vascular wall inflammation and impaired extracellular matrix remodeling. Together, GzmB is an important contributor to age-dependent impaired PI healing.

## Introduction

Pressure injuries (PIs), also known as bedsores or pressure ulcers, are areas of localized damage to the skin and underlying tissue due to pressure, shear or friction. Ischemia and inflammation are hallmarks of PI pathogenesis, which frequently occur over bony prominences where bone puts pressure on skin for a prolonged period. Aging contributes to poor dermal perfusion, skin frailty, loss of skin elasticity and firmness. Combined with a more sedentary lifestyle, aging provides an ideal scenario for PI development (reviewed in ref. ^1^). This may also be exacerbated by reduced sensory perception, poor nutrition, and urinary and fecal incontinence^2^. PI in aged skin is more likely to display persistent inflammation, increased wound severity, and delayed healing. Standard care typically involves patient rotation in order to offset pressure every 2 h to encourage adequate perfusion. However, for a variety of reasons, adequate care is often not received^3^.

PI severity is defined by both injury depth and the type of tissue affected. Stage I PIs present with non‐blanchable erythema. In stage II PIs, there is a loss of epidermis and damage penetrating into the dermis. In stage III PIs the wound penetrates through to the subcutaneous tissue, while in stage IV, there is further wound penetration involving muscle necrosis and potential joint/tendon damage. Once PIs progress to past stage 2 they become progressively more difficult to treat, are debilitating, susceptible to infection, often require surgical intervention, and there is increased chance of wound recurrence. While prevention is the first line of defense, effective therapeutic approaches to address early stage PIs would provide an enormous benefit to those affected.

Granzyme B (GzmB) is a serine protease traditionally identified as being involved in lymphocyte-mediated apoptosis. Secreted in combination with the pore-forming protein, perforin, which facilitates entry into the target cell, GzmB initiates apoptosis^4^. However, during effector–target cell engagement, approximately one-third of GzmB disperses away from the immunological synapse and into the extracellular space^5^. Further, in a more recent shift in paradigm, other cell types can also express and secrete GzmB, including B cells, basophils, mast cells, neutrophils, dendritic cells, and keratinocytes (reviewed in ref. ^6^), with many of these cells expressing GzmB independently of perforin and/or lacking the capacity to form immunological synapses with target cells. This presence of functionally active extracellular GzmB released by both immune- and non-immune-cells therefore suggest that this protease exerts additional roles independent of apoptosis.

GzmB is expressed at low levels in healthy tissue, but is dramatically upregulated in aged skin^7,8^ and diabetic ulcers^9^. Multiple validated substrates of GzmB have been described in skin including vitronectin^10^, decorin^11^, and fibronectin^10^. Perforin-independent GzmB-mediated cleavage of these and other proteins has been linked to impaired collagen remodeling^12^, enhanced extracellular matrix (ECM) degradation^7,10^, and increased endothelial permeability^13^.

GzmB progressively accumulates in conditions associated with age and chronic inflammation, suggesting a role in inflammaging (chronic, low-grade inflammation associated with aging)^6,14^. The upregulation of GzmB in response to aging and disease, combined with the ability to cleave a range of extracellular substrates in skin, suggests that GzmB contributes to chronic disease and impaired wound healing^15^. The role of GzmB in disease appears to be context-dependent, and varies according to the cell source and where GzmB accumulates in the skin (reviewed in ref. ^6^). Further, GzmB retains its proteolytic activity in human plasma^16^ and no endogenous inhibitors of extracellular GzmB have been identified to date. As such, GzmB may act unimpeded as it accumulates during aging in damaged tissues, especially those of a chronic nature.

Apolipoprotein E is one of the most studied genes in longevity research (reviewed in ref. ^17^), with apolipoprotein E knockout (ApoE−/−) mice providing an invaluable model to study aging. Aged ApoE−/− fed a high-fat diet (HFD) display skin thinning, loss of thick fiber formation and collagen density, and elastin abnormalities, while also showing elevated GzmB detection^8^. In addition to displaying skin fragility^8^, ApoE−/− mice also exhibit impaired wound healing^15^. ApoE−/− mice are also an established model of atherosclerosis, with a history of atherosclerosis predictive of PI, while a primary diagnosis of atherosclerosis is associated with a greater likelihood of developing a hospital acquired PI^18^. Critically in the context of PI, aged ApoE−/− mice fed an HFD have impaired tissue perfusion^19^. Thus, the skin of these mice is highly likely to be susceptible to GzmB in the context of ischemia–reperfusion injury. As aged wild-type mice exhibit minimal atherosclerosis, ApoE−/− mice are a relevant model to study GzmB and its role in PI in the context of aging in humans.

In the present study, GzmB was elevated in the skin of human and mouse PI tissues. In young healthy mice, GzmB did not impact PI severity or healing. In aged ApoE−/− mice, GzmB contributed to increased disease severity, functioning to increase inflammation, elevate vascular micro-hemorrhage, increase ECM degradation and also fibrotic activity.

## Results

### GzmB elevated in human PI

**Fig. 1:**
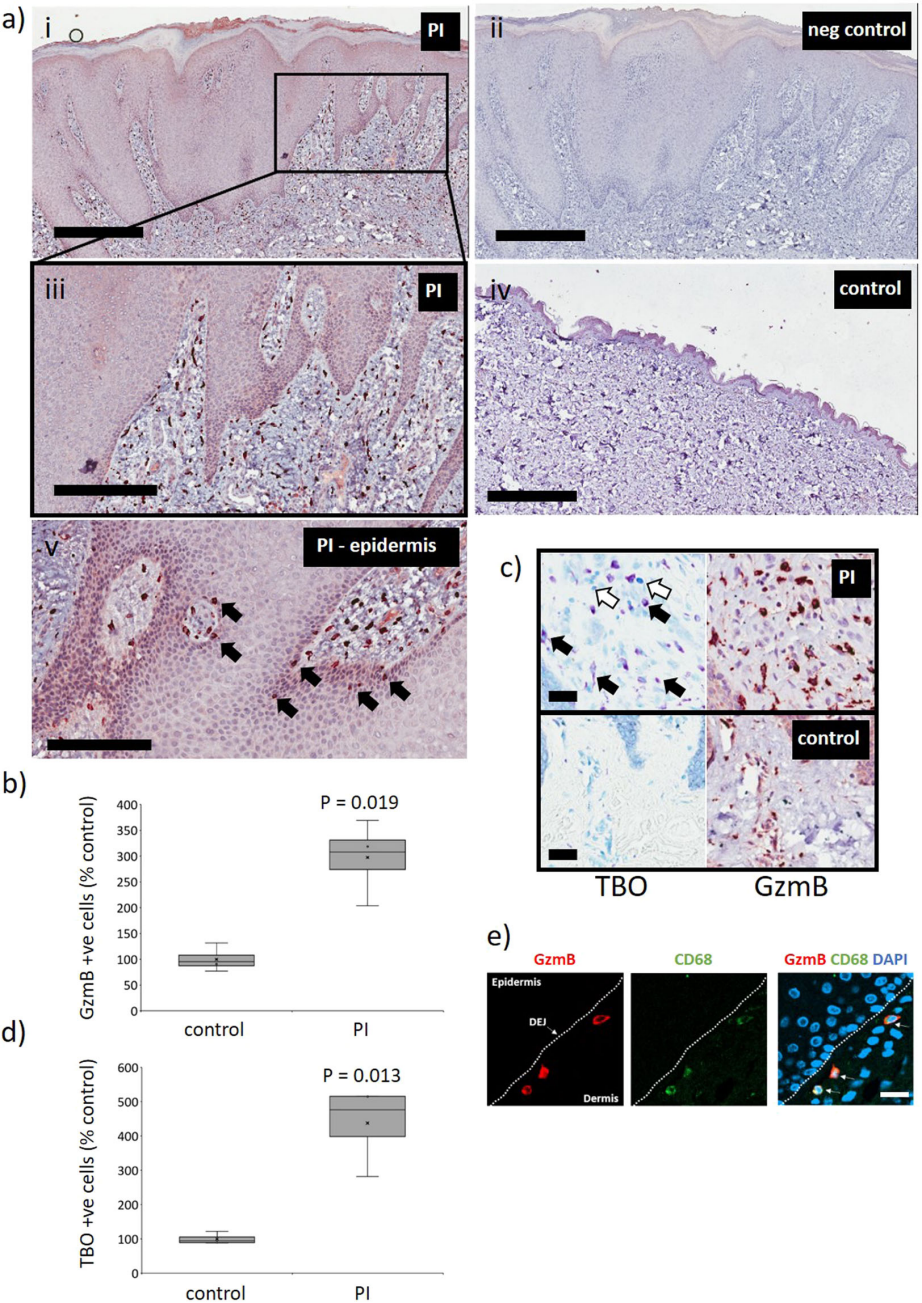
Elevated GzmB in human PI. **a** GzmB immunohistochemistry in human PI and unwounded control skin. Scale bars = 600 µm (i, ii, iv), 300 µm (iii), and 200 µm (v). **b** Quantification of GzmB+ cells. Data presented in box and whisker plot as the percentage increase of GzmB+ cells per area compared to the unwounded control, *n* = 3 samples per group. **c** TBO (mast cell marker) stain and GzmB immunohistochemistry on sequential tissue sections. Black arrows indicate representative TBO+ cells while black arrows indicate representative GzmB+/TBO− cells. Scale bars = 30 µm. **d** Quantification of TBO+ cells. Data presented as the percentage increase of TBO+ cells per area compared to the unwounded control, *n* = 3 samples per group. **e** GzmB (red) and CD68 (green, macrophage marker) and DAPI (blue) immune-fluorescence (IF) of human PI. Dotted line is dermal–epidermal junction (DEJ). Scale bars = 20 µm.

GzmB expression was investigated by immunohistochemistry in human PI tissue and control skin (Fig. 1a). PI samples, obtained from patients ranging from 53 to 77 years of age (*n* = 3), were clinically identified as stage 3 in severity (Table 1). GzmB+ cells were significantly elevated in human PIs compared to unwounded skin controls (ages 44–63), where minimal numbers were observed (*P* = 0.019; Fig. 1a, b). GzmB+ cells were distributed throughout the dermis, including in regions of inflammatory cell infiltrate, while a small number was also identified in the epidermis.

**Table 1:** PI patient data.

Sample	Stage	Wound age (months)	Sex	Infection	Patient information	Patient age	GzmB (pg/mL)
PI Tissue 1	3/4	NA	M	NA	First nations, type 2 diabetes	54	ND
PI Tissue 2	3/4	NA	M	NA	Caucasian	66	ND
PI Tissue 3	3/4	NA	M	NA	Caucasian	73	ND
Cont Tissue 1	–	NA	M	N	Caucasian	44	ND
Cont Tissue 2	–	NA	M	N	Unreported	52	ND
Cont Tissue 3	–	NA	F	N	Caucasian	63	ND
WF 1	3	>36	M	Y	Antibiotic/antimicrobial therapy	40	125
WF 2	3	6	M	Y	–	44	0
WF 3	3	10	F	Y	–	45	4
WF 4	3	>12	M	Y	Cannabis smoker	49	215
WF 5	3	6	F	Y	–	52	0
WF 6	3	8	F	Y	–	53	299
WF 7	3	18	M	Y	Antimicrobial dressing	58	116
WF 8	3	6	M	Y	–	60	0
WF 9	3	NA	M	Y	–	75	206
WF 10	3	6	M	Y	Antibiotic therapy	84	0
WF 11	4	13	M	Y	Antimicrobial dressing, Cannabis smoker	27	260
WF 12	4	15	NA	N	–	65	2537
WF 13	4	NA	F	Y	Smoker	–	911

*NA* not available, *ND* not determined.

GzmB is elevated in UV damaged skin, with mast cells identified as being in part responsible for GzmB expression^7^. In human PI samples, toluidine blue (TBO, a mast cell marker)-positive cells were elevated compared to healthy skin controls (*P* = 0.013), with all mast cells analyzed observed to express GzmB, and accounting for the majority of total GzmB+ cells in the wounded tissue (Fig. 1c, d). GzmB was also expressed to a lesser extent by non-mast cell population/s (Fig. 1c). CD68+ (macrophage/monocyte) cells exhibited GzmB immune-positivity (Fig. 1e). Notably, not all CD68+ cells were GzmB positive, suggesting a specific sub-population of CD68+ cells, one with a tissue localization in the dermis but close to the dermal–epidermal junction. In PI, CD68+ cells accounted for approximately <5% of total cells expressing GzmB. Further investigation is required to identify other cell source(s) of GzmB.

Wound fluid samples were obtained from donor individuals with stage 3 and 4 PIs and assessed for GzmB levels by enzyme-linked immunosorbent assay (ELISA) (*n* = 13, Table 1). Wound fluid from stage 4 PIs had overall higher concentration of GzmB than stage 3. Fluid from wounds open for greater than 10 months exhibited higher concentrations of GzmB compared to that from wounds open less than 10 months, although there was an insufficient number of samples in each group to determine significance. Together with the histology data, the observed increase in GzmB in stage 4 and slow-healing PI wounds suggests GzmB concentration increases with PI severity.

### Cutaneous GzmB is elevated in both aged humans and ApoE−/− mice fed on an HFD

**Fig. 2:**
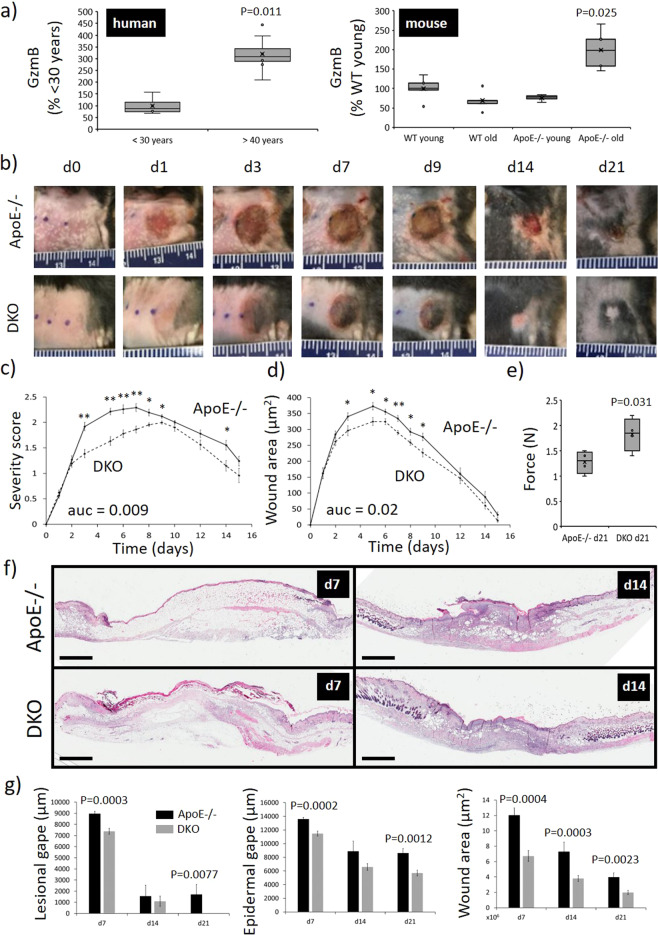
Reduced wound severity in DKO mice PI. **a** Relative GzmB expression in human and mouse skin controls. **b** PI photos. **c** PI Severity score. **d** Macroscopic measure of wound area. Data in **c**, **d** presented as mean ± SEM, *n* = 6. *P < 0.05, **P < 0.005. **e** Minimum wound breaking force at days 21, *n* ≥ 4 mice per group. **f** Representative images of H&E-stained mouse PI tissue. Scale bars = 1.5 mm. **f** Wound measurements calculated from the H&E-stained PI tissue. Presented as mean ± SEM, *n* = 6.

GzmB was quantified in a small group of unwounded control skin derived from human donors, ranging in age from 21 to 70 (Supplementary Table 1), displaying increased GzmB+ cells with age and showing a threefold increase in samples >40 compared to <30 years of age (*P* = 0.011) (Fig. 2a). GzmB was then quantified in unwounded skin from WT mice and compared to that from ApoE−/− mice, a model of premature skin aging. In young mice (8 weeks), no difference in GzmB+ cells between WT and ApoE−/− mice skin was observed, with chronologically aged WT mice (52 weeks, considered middle aged) also showing similar amounts of GzmB expression (Fig. 2a). In contrast, prematurely aged ApoE−/− mice (38 weeks), which involved being fed an HFD for 30 weeks (now referred to as ApoE−/− mice) showed a greater than twofold increase in GzmB expression compared to young ApoE−/− mice (*P* = 0.025), a similar increase to that observed in the aged human skin.

### GzmB contributes to increased PI severity in ApoE−/− mice fed on an HFD

Despite PIs being an enormous socioeconomic burden to the healthcare system, there remains no universally accepted animal models for the study of such wounds, nor the impact of aging and other co-morbidities such as atherosclerosis on the healing of such wounds. Repeated cycles of ischemia and reperfusion (I/R) are an important contributor to the initiation and severity of PI wounds. In patients, PIs usually occur over bony prominences whereby the pressure can cut off circulation, while reperfusion leads to a burst of reactive oxygen intermediates followed by endothelial dysfunction and the release of pro-inflammatory cytokines^20^. Adapted from a previously reported technique^21^, a non-invasive mouse model incorporating multiple rounds of I/R using magnets was therefore adopted for this study (Supplementary Fig. 1).

When 10-week-old WT C57Bl/6 mice were subjected to I/R, PIs developed that approached stage 2 in severity (Supplementary Fig. 2), as determined by a severity grading scale which provides a visual measure of skin loss, injury depth, and erythema (Supplementary Table 2). There was pronounced erythema and some loss of epidermis, but without evidence of fat/muscle damage. Aged ApoE−/− were also subjected to I/R. Magnet exposure in these mice led to larger and more severe wounds (Fig. 2b–d) with more extensive tissue damage than observed in WT mice including injury within deeper tissue layers, such as the panniculus carnosus (Fig. 2f). Wound severity scores in ApoE−/− mice peaked approximately 30% higher than WT mice (scores of 2.3 compared to 1.8 at d7). Histologically, injuries from both WT and ApoE−/− mice displayed evidence of a hyperproliferative epidermis and increased inflammatory cell infiltrate (Supplementary Fig. 2 and Fig. 2e). I/R-mediated injury therefore appears to induce PI similar to those observed in humans, while producing more severe PIs in the aged mouse model.

To identify a causative role for GzmB, PIs were induced in 10-week-old GzmB−/− mice and compared to age-matched WT mice. There was no difference in weight between WT and GzmB−/− mice post-initiation of I/R, while macroscopically, wound severity and wound area displayed no difference (Supplementary Fig. 2). These findings correlated with a lack of difference in lesion gape (length of wound region where epidermis is completely destroyed), epidermal gape (length of wound region combining epidermal gape and areas of hyperproliferative epidermis), wound margin (total width of wound), and wound area (total area of wound) as measured from H&E-stained wound tissue (Supplementary Fig. 2). These data suggest GzmB to have no impact on PI development and severity in the skin of otherwise healthy, young mice.

PI severity was compared between ApoE−/− and HFD GzmB/ApoE double knockout (DKO) mice (now referred to as DKO mice). Thirty weeks post HFD, and prior to I/R injury, no difference in weight was identified between the two mouse groups (Supplementary Fig. 3). In response to PI induction, there were no apparent differences in the overall health of the mice, and with no mice requiring euthanasia. Mice from both groups lost approximately 7% body weight after 7 days post-initiation of I/R, but there was no difference between the ApoE−/− and DKO mice (Supplementary Fig. 3). Wound severity scores were reduced in DKO compared to ApoE−/− mice (area under curve (auc), *P* = 0.009) (Fig. 2b, c). Wound area was also reduced in DKO compared to ApoE−/− mice (auc, *P* = 0.02) (Fig. 2b, d). Wound tensile strength was improved in DKO compared to ApoE−/− at d21 (*P* = 0.031; Fig. 2e). As measured from H&E-stained tissue sections, the wound margin (*P* ≤ 0.042, data not shown) and wound area (*P* ≤ 0.0023) of DKO PI were reduced at all time points post-initiation of I/R compared to the ApoE−/− (Fig. 2f, g). Lesional gape (*P* = 0.0003 at d7 and *P* = 0.0077 at d27) and epidermal gape (*P* = 0.0002 at d7 and *P* = 0.0012 at d27) were also reduced in DKO compared to ApoE−/− PI. Together, exposing mouse skin to repeated rounds of I/R induced wounds similar to the development of human PI, while GzmB contributes to wound severity and repair in aged but not young mice.

### Elevated GzmB in mouse PI

**Fig. 3:**
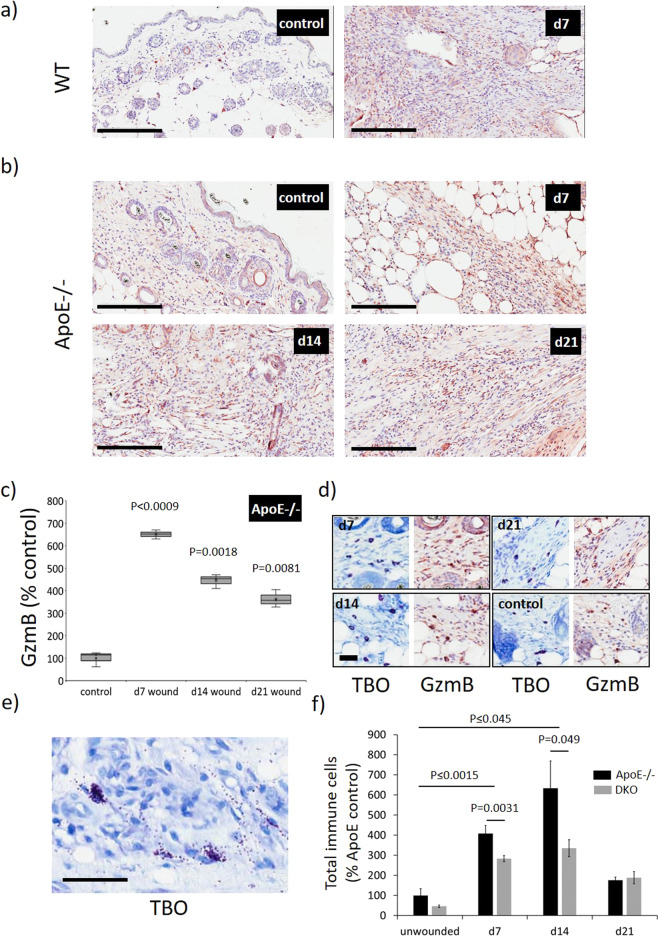
Elevated GzmB in ApoE−/− mice PI. GzmB immunohistochemistry in WT (a) and ApoE−/− (**b**) mouse PI and unwounded skin controls. Scale bars = 200 µm. **c** Quantification of GzmB+ cells in ApoE−/− PI and unwounded skin controls. Data presented as the percentage increase of GzmB+ cells per area compared to the unwounded control; *n* = 4 samples per group. Data analyzed by two-way ANOVA with Bonferonni post-test. **d** TBO (mast cell) stain and GzmB immunohistochemistry on sequential tissue sections. Scale bar = 30 µm. **e** High magnification image displaying mast cell degranulation in mouse PI tissue at d14 post-injury. Scale bar = 50 µm. **f** Quantification of total inflammatory cell infiltrate in mouse PI as measured from H&E-stained tissue sections. Data analyzed by two-way ANOVA with Bonferonni post-test and presented as total immune cells per unit area as a percentage of the unwounded ApoE−/− control, mean ± SEM (*n* = 6 per group).

To identify whether GzmB levels and/or tissue distribution may account for the difference in PI severity between aged and young mice, GzmB immunohistochemistry was performed. Following I/R-mediated injury, GzmB+ cells were elevated in the wound margin of both WT (Fig. 3a) and ApoE−/− PIs (*P* > 0.0081; Fig. 3b, c) compared to unwounded skin. At d7, the amount of GzmB+ cells in the dermis of ApoE−/− PI and WT PI was similar, with GzmB+ cells mainly localizing to regions of inflammatory cell infiltrate. However, in ApoE−/− PI, with deeper wounds than WT PI, GzmB+ cells were also identified within regions of fat and the panniculus carnosus. TBO+ cells were elevated in ApoE−/− PI compared to unwounded control skin, and similarly to human PI tissue, were all found to express GzmB (Fig. 3d), suggesting mast cells as a major source of GzmB in I/R-mediated PI. These mast cells had both intact and degranulated phenotypes, in which GzmB appeared to be released from the cell (Fig. 3e). Similar to human PI, a smaller population of CD68+ cells were found to express GzmB (Supplementary Fig. 4).

### GzmB contributes to increased inflammation in mouse PI

To investigate a mechanistic role for GzmB, epidermal proliferation, apoptosis, and inflammation were measured in aged mice PI. Epidermal proliferation, as measured by Ki67 and PCNA, and apoptosis, as measured by TUNEL, were elevated in PI compared to skin controls, but no difference was identified between aged ApoE−/− and DKO mice (data not included). In aged mice, total inflammatory cell numbers were elevated in d7 and d14 PI tissue compared to unwounded controls (*P* ≤ 0.045; Fig. 3f). DKO mice PI displayed reduced inflammation compared to ApoE−/− mice (*P* ≤ 0.0031 at d7, *P* ≤ 0.049 at d14). To identify specific inflammatory processes associated with this reduction, a panel of cytokines/chemokines were screened in PI wound tissue extract using a proteome profiler antibody array. In d7 ApoE−/− PI, soluble intercellular adhesion molecule-1 (sICAM-1), macrophage inflammatory protein-2 (MIP-2), interleukin-1ra (IL-1ra), MIP-1α, and C5/C5a were the most abundant, while sICAM-1 and IL-1ra were additionally abundant at d14 (Supplementary Fig. 5). In d7 DKO PI, sICAM-1 was reduced compared to ApoE−/− PI, with marginal increases observed in IL-1ra, MIP-1α, and MIP-2 (Supplementary Fig. 5). sICAM-1, IL-1β, IL-16, TIMP-1, and TREM-1 were all reduced in d14 DKO PI compared to ApoE−/− PI, while the expression of IL-1α, MIP-1α, and MIP-2 was increased.

### GzmB contributes to increased microvascular permeability and micro-hemorrhage in mouse PI

**Fig. 4:**
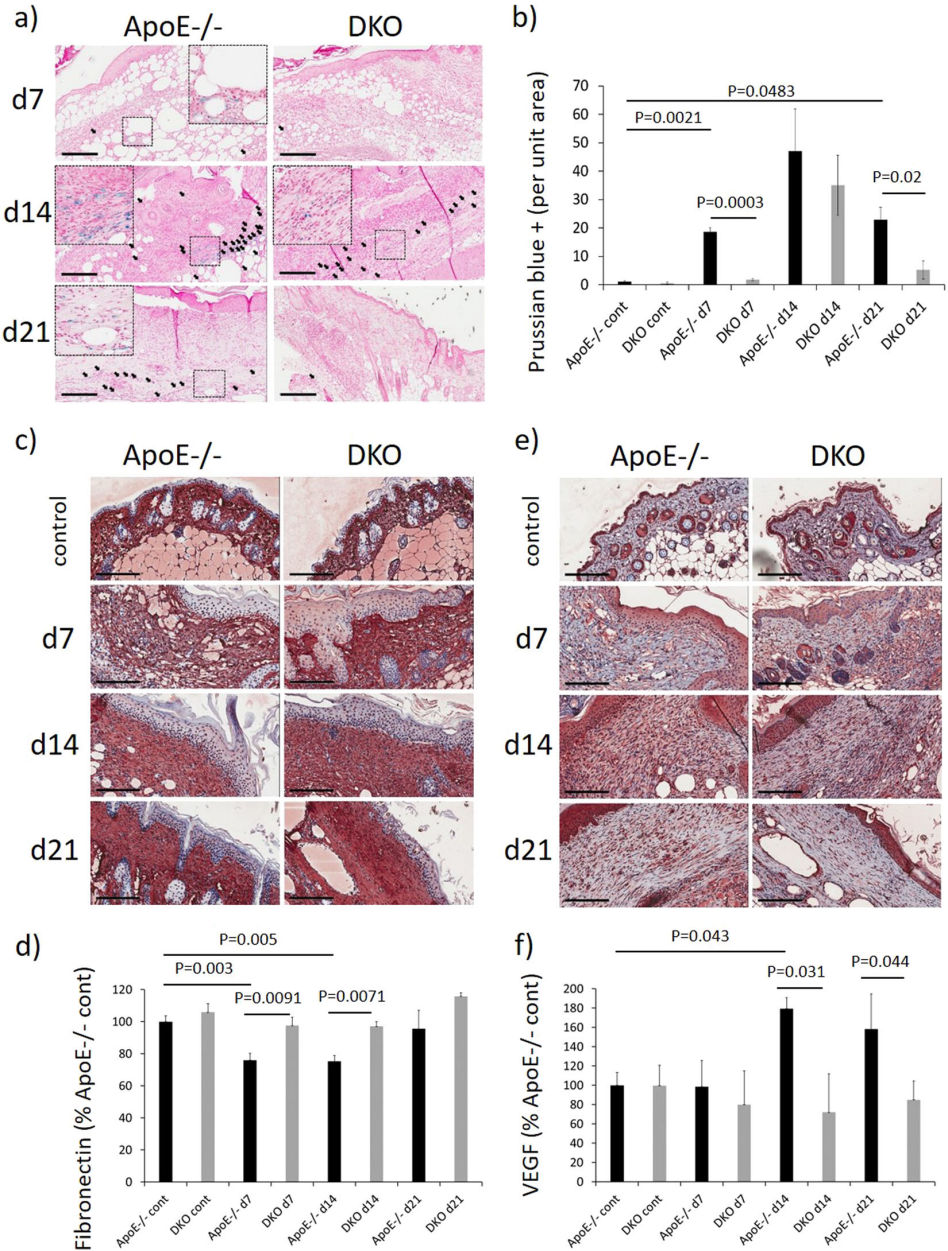
Reduced micro-hemorrhage in DKO mice PI. **a** Representative images of Prussian blue staining (**a**), fibronectin (**c**), and VEGF (**e**) immunohistochemistry in ApoE−/−, DKO mice PI and unwounded controls. Scale bars = 300 µm (**a**) and 200 µm (**c**, **e**). Quantification of Prussian blue (**b**), fibronectin (**d**), and VEGF (**e**). Data analyzed by two-way ANOVA with Bonferonni post-test and presented as Prussian blue staining per unit area (**b**), fibronectin (**d**), or VEGF (e) staining intensity in the dermis as a percentage of ApoE−/− unwounded control skin, mean ± SEM, *n* = 6.

sICAM-1 showed the most abundant expression of all markers evaluated in the cytokine/chemokine screening assay, while also displaying the most pronounced reduction in DKO compared to ApoE−/− PI. sICAM-1 is a potential biomarker of endothelial activation^22^ and vascular wall inflammation^23^. Serum sICAM-1 levels are also associated with the presence of cerebral micro-bleeds and increased risk of hemorrhagic transformation in ischemic stroke patients^24^. ICAM-1 ELISA, which detects both membrane-bound ICAM-1 and sICAM-1, displayed elevated sICAM-1 in ApoE−/− compared to DKO PI samples at d14 (*P* = 0.045) (Supplementary Fig. 5), supporting GzmB as possibly contributing to elevated sICAM-1 in PI and therefore increased microvascular permeability. Perl’s Prussian blue staining, which measures interstitial hemosiderin deposition, an indication of micro-hemorrhage and increased microvascular permeability, showed ApoE−/− mice wounds to have significantly elevated extravasation of hemosiderin compared to equivalent tissue in DKO mice (*P* = 0.0003 at d7 and *P* = 0.02 at d21; Fig. 4a, b). Extravasation was predominantly localized to the dermis in close proximity to the dermal white adipose tissue layer.

Fibronectin is a validated cleavage target of GzmB^10^. It was previously reported that GzmB-mediated fibronectin cleavage releases VEGF thereby inducing vascular permeability^25,26^. In ApoE−/− mouse PI tissue, fibronectin was reduced at d7 (*P* = 0.003) and d14 (*P* = 0.005) post-induction of I/R compared to unwounded control skin, while DKO displayed significantly more fibronectin than ApoE−/− at these two time points (*P* = 0.0091 at d7 and *P* = 0.0071 at d14) (Fig. 4c, d). VEGF, also elevated in ApoE−/− PI tissue compared to unwounded control skin (*P* = 0.043 at d14), was significantly elevated in ApoE−/− compared to DKO at d14 (*P* = 0.031) and d21 (*P* = 0.044; Fig. 4e, f).

As vascular wall inflammation and microvascular permeability was reduced in DKO compared to ApoE−/− PI, perfusion within the wound may also be affected. Tissue perfusion was therefore evaluated in the injured skin using Doppler (Supplementary Fig. 3). Unwounded DKO mice skin displayed elevated perfusion compared to ApoE−/− mice (*P* < 0.005), suggesting ApoE−/− mice to have impaired microcirculation similar to that commonly observed in the elderly. I/R immediately led to a drop in tissue perfusion in the DKO mice, which was not as pronounced in the ApoE−/− mice, both plateauing at d3 (corresponding to d1 post-initiation of I/R), and suggesting similar depth of injury. There was no difference in perfusion between ApoE−/− and DKO mice wounds from d2 to d9. Perfusion was unable to be determined for latter time points as the development of scabs on the wounds prevented Doppler penetration.

### Decorin is cleaved by GzmB and impairs collagen organization in mouse PI

**Fig. 5:**
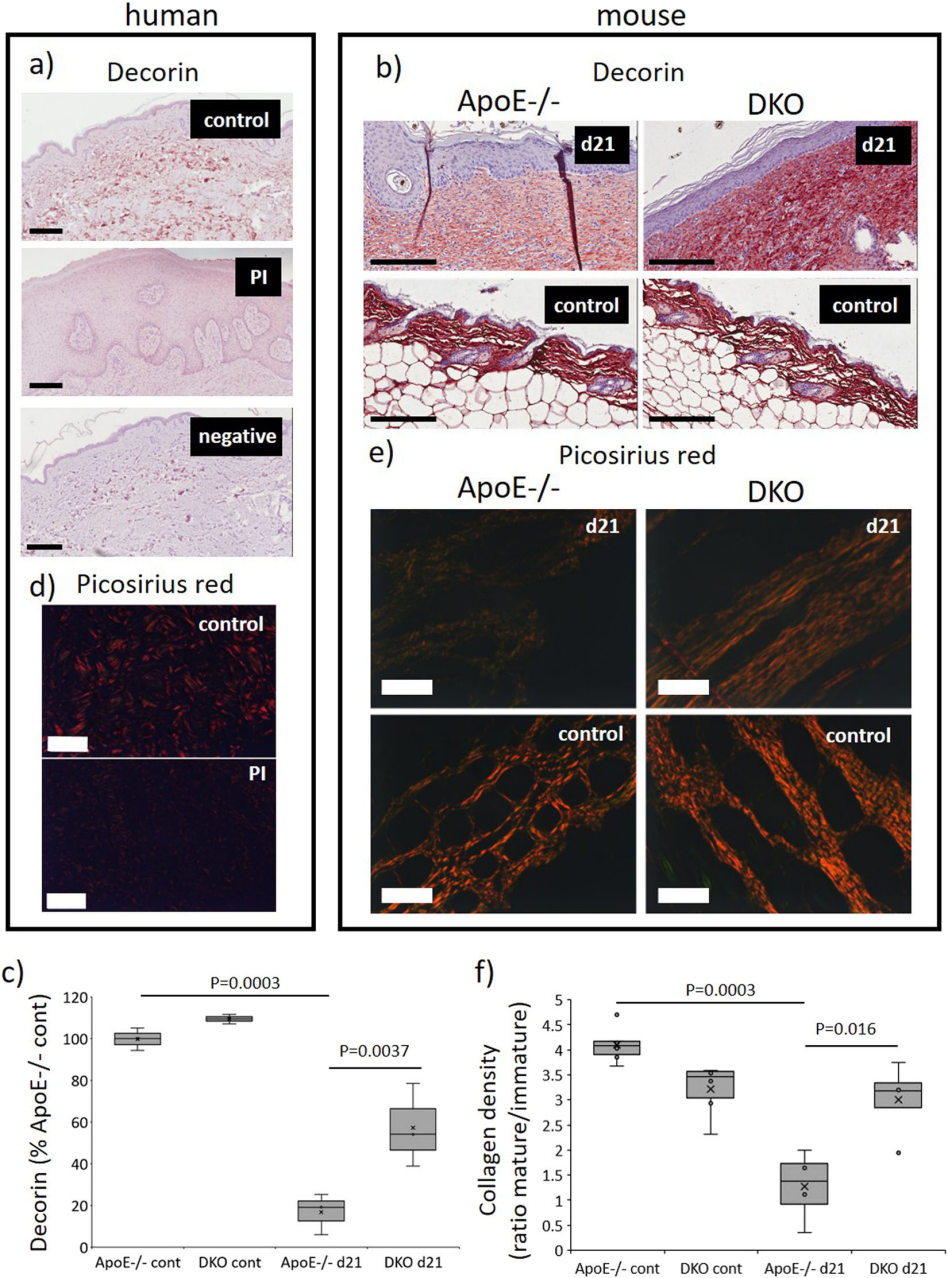
Reduced decorin intensity and improved collagen maturation in DKO mice PI. Representative images of decorin immunohistochemistry (**a**) and Picosirius red (**d**) in mouse PI at d21 post-initiation of I/R. Scale bar = 300 µm (**a**) and 200 µm (**d**). Quantification of decorin (**c**) and Picosirius red (**f**). Data in **c**, **f** analyzed by two-way ANOVA with Bonferonni post-test and presented as **c** intensity per unit area as the percentage of ApoE−/− control and **f** the ratio of mature (red) to immature (green) collagen, *n* = 4 samples per group. Representative images of decorin immunohistochemistry (**b**) and Picosirius red (**e**) in human PI and unwounded control skin. Scale bar = 200 µm (**b**, **e**).

Decorin is a validated cleavage target of GzmB^27^. In human PI tissue, there was an almost complete absence of decorin within the dermis (Fig. 5a), suggesting decorin may be acted upon proteolytically. In d21 ApoE−/− PI, decorin was significantly reduced in the dermis compared to unwounded skin controls, agreeing with the human data (*P* = 0.003; Fig. 5b, c). Significantly more decorin could be detected in the dermis of DKO than ApoE−/− mice (*P* = 0.0037), suggesting GzmB mediates decorin cleavage in PI.

Decorin cleavage in the skin disrupts fibrillogenesis and impairs collagen organization^8,11,27^; thus, collagen was investigated in PI tissue undergoing the remodeling stage of wound repair. Picosirius red staining under polarized light showed greatly reduced collagen intensity in human PI samples compared to age-matched unwounded skin controls, which displayed thick collagen bundles (Fig. 5d). Mouse PI tissue at d21 post-initiation of I/R also showed reduced collagen density compared to unwounded skin, and similarly to decorin detection, DKO wound tissue showed higher Picosirius red than observed in the ApoE−/− mice (*P* = 0.0011; Fig. 5e, f). Together, GzmB-mediated cleavage of decorin in PI contributes to impaired collagen organization during tissue remodeling.

### GzmB contributes to increased fibrotic activity in mouse PI

**Fig. 6:**
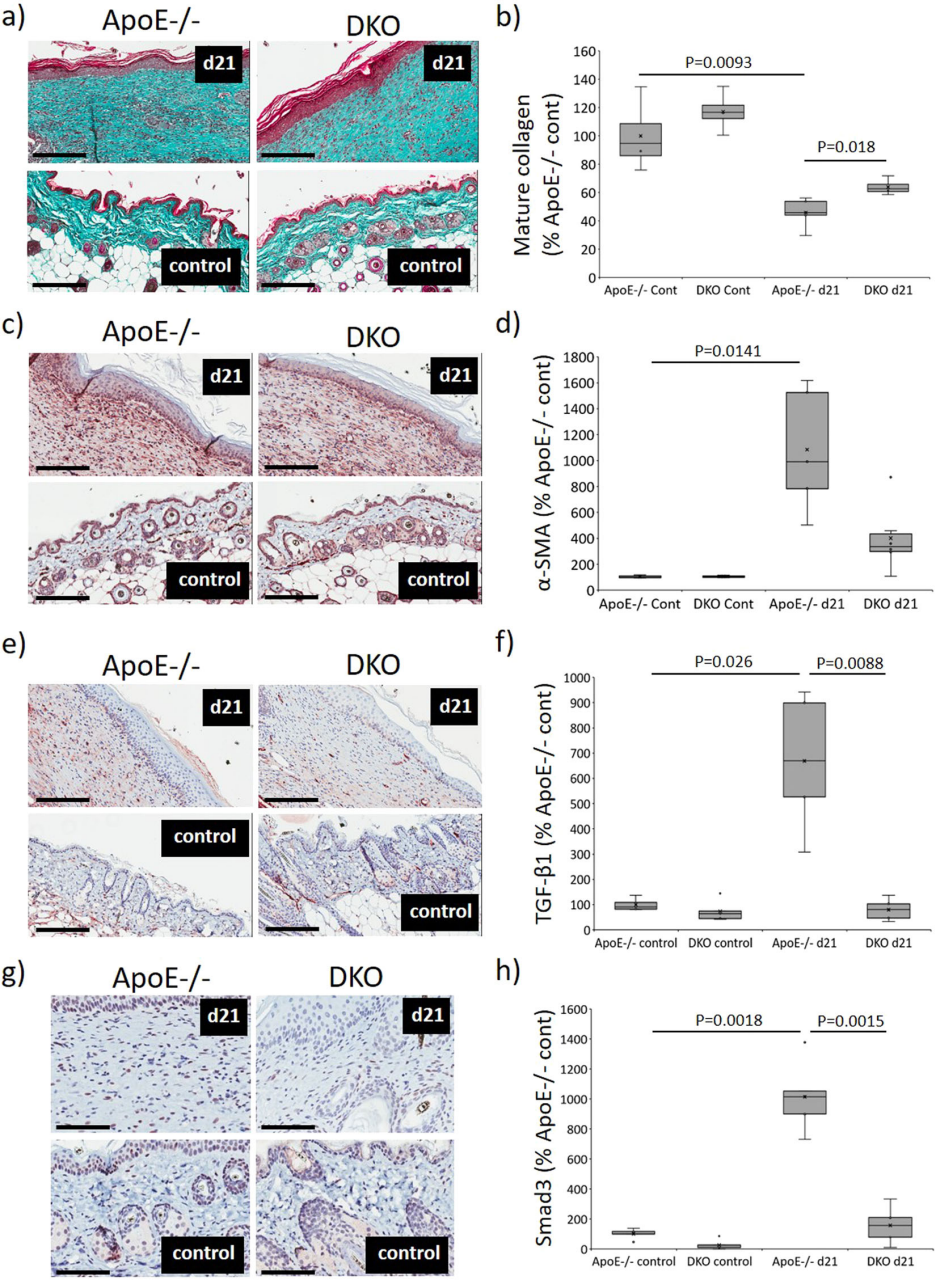
Reduced fibrotic activity in DKO mice PI. Representative images of Masson’s trichrome (**a**), α-SMA immunohistochemistry (**c**), TGF-β1 (**e**), and Smad3 (**g**) in mouse PI at d21 post-initiation of I/R. Scale bars = 200 µm (**a**, **c**, **e**) and 100 µm (**g**). Quantification of Masson’s trichrome (**b**), α-SMA (**d**), TGF-β1 (**f**), and Smad3 (**h**). Data analyzed by two-way ANOVA with Bonferonni post-test and presented as a percentage of ApoE−/− control, *n* = 4 samples per group.

Collagen deposition was further investigated in mice PI, focusing on the remodeling phase of wound repair. As demonstrated by Masson’s trichrome staining of d21 PI tissue, increased mature collagen was detected in DKO PI compared to ApoE−/− wounds (*P* = 0.018; Fig. 6a, b). Alpha-smooth muscle actin (α-SMA) intensity was threefold higher in ApoE−/− than DKO PI wounds (*P* = 0.0141; Fig. 6c, d), suggesting elevated recruitment of myofibroblasts. Transforming growth factor-β1 (TGF-β1) was elevated in the d21 ApoE−/− PI compared to both unwounded ApoE−/− skin (*P* = 0.026) and DKO PI (*P* = 0.0088; Fig. 6e, f). Finally, there was increased expression of Smad3 in d21 ApoE−/− PI compared to unwounded controls (*P* = 0.0018), while no change was observed in d21 DKO PI (Fig. 6g, h).

## Discussion

GzmB is elevated in response to multiple age-related and autoimmune diseases (reviewed in ref. ^6^) and inhibition of GzmB attenuates premature skin aging and improves collagen organization^7,8,28^. Given the link between increased age and PI pathogenesis, the role of GzmB in PI was investigated in mice and humans. GzmB was dramatically elevated in advanced chronic human PI tissue, localizing to inflammatory cell infiltrate in the dermis while also minimally evident in the epidermis. In a limited sample of PI wound fluids, GzmB concentration was higher in the most severe wounds, therefore suggesting this serine protease contributes to disease pathogenesis.

Traditionally, the cellular expression of GzmB was reported exclusively in NK and cytotoxic T-lymphocytes, with these functioning to induce perforin-dependent cytotoxicity. However, many of these studies were performed prior to our current understanding of the extracellular roles for GzmB and/or do not acknowledge the fact that GzmB can be expressed in other cell types, including those important in wound healing (reviewed in ref. ^6^). In the current study, mast cells were identified as a major source of GzmB in PI tissue. Mast cell-derived GzmB expression is also observed in intrinsically aged skin from human donors^29^ and mice exposed to chronic, low-dose UV irradiation^7^. Saliently, dermal mast cells express and secrete GzmB^7,30^, while cytotoxic T-lymphocytes showed no GzmB in intrinsically aged skin^29^. Providing a smaller overall contribution to expression in PI, GzmB also appeared to be expressed by a subset of CD68+ cells. Activated monocytes have also been previously identified as expressing and importantly secreting GzmB^31^. GzmB therefore appears to contribute extracellularly rather than by inducing cytotoxicity in PI pathogenesis.

Despite being elevated in PI, a causative role for GzmB is not known. Animal studies were therefore performed in both young and aged mice subjected to multiple rounds of I/R injury. This non-invasive approach showed the major hallmarks of human PI, including elevated inflammatory cell recruitment, tissue damage deep into the dermis, and hyperproliferative epidermis. Moreover, GzmB expression was similar to that observed in human PI tissue, with a similar tissue distribution within the dermis and predominately expressed by mast cells. I/R injury to the skin of mice therefore provides a useful model to study GzmB and its role in PI.

In the PI model using younger mice, no difference in lesion severity and wound healing between GzmB−/− and WT mice was observed. However, PI is more prevalent in elderly populations, which exhibit skin frailty, skin thinning, loss of dermal collagen density, impaired tissue perfusion, and elevated cutaneous expression of GzmB. Thus, when PI was induced in aged ApoE−/− mice, wounds were more severe that observed in young WT mice. Saliently, in DKO mice PIs, wound severity was reduced, there was more rapid wound closure and increased tensile strength compared to ApoE−/− mice, suggesting GzmB as contributing to disease pathogenesis in this model of cutaneous aging.

Unwounded control skin from the prematurely aged ApoE−/− mice displayed elevated GzmB expression compared to both young ApoE−/− and young WT mice. Notably, GzmB positivity was not elevated in unwounded skin from aged WT mice. However, these 52-week-old mice are only considered to be middle aged. Two-year-old mice, more reflective of old age, may be more likely to display increased GzmB expression and are therefore a focus of future study. Together, middle-aged WT mice are not an appropriate model for human aging in the context of GzmB.

I/R injury is considered as a significant factor in the development of PI. Since PIs show fundamental differences in disease etiology compared to other injuries studied in the wound healing field, including excisions and burns, we decided to investigate specific components of disease that were more unique to PI, namely changes to tissue vascularity and vessel extravasation. sICAM-1, elevated in PI tissue in the presence of GzmB, has been identified as a marker of vascular inflammation^23^. In the current study, dermal Prussian blue staining was elevated in the presence of GzmB, suggesting GzmB contributes to increased vascular permeability and micro-hemorrhage in the context of PI.

GzmB-mediated fibronectin cleavage has been shown to release VEGF from fibronectin resulting in activation of VEGFR2 and increased vascular permeability^32^. Fibronectin exposed to GzmB also induces the release of VEGF from endothelial cells in culture^32^. Moreover, GzmB-mediated fibronectin cleavage in ApoE−/− excisional wounds has been proposed to delay excisional wound healing^15^. In the current study, fibronectin was reduced in PI tissue in the presence of GzmB, suggesting cleavage, while there was a corresponding increase in VEGF detection. GzmB therefore contributes to vessel damage in response to I/R injury, and therefore provides a contributing role to PI pathogenesis.

Several studies have proposed a role for GzmB in fibrosis (reviewed in ref. ^6^). GzmB expression is upregulated in the infiltrating lymphocytes of lung tissue from patients with idiopathic pulmonary fibrosis compared with normal lung parenchyma^33^. In a cardiac model of fibrosis, genetic GzmB deficiency led to reduced angiotensin II-induced cardiac hypertrophy and less fibrotic activity^13^. In the current analysis of PI tissue during the late-stage remodeling phase of wound repair, DKO mice displayed reduced deposition of immature collagen and decreased detection of myofibroblasts. Additional markers of fibrosis and scarring, namely TIMP-1 and TREM-1 were also identified as being elevated in the presence of GzmB. Together, this suggests that GzmB appears to have an important contribution to the development of fibrosis in skin following exposure to I/R.

GzmB-mediated cleavage of decorin has been demonstrated to impair healing^15^, including in a skin aging mouse model^8^. Leading to disrupted fibrillogenesis and impaired collagen organization, GzmB-mediated decorin cleavage releases active TGF-β1 (ref. ^11^), which stimulates myofibroblast formation, providing a mechanistic link between GzmB and fibrosis. Decorin is reduced in fibrotic tissues in skin and other organs, leading to the disorganized collagen that characterizes these lesions. The administration of recombinant decorin to wounds in animal models reduces fibrosis^34^. In the current study, PI wound tissue in the remodeling phase of wound repair displayed reduced decorin, impaired collagen organization, and a corresponding increase in α-SMA and TGF-β1, while in the absence of GzmB these effects were mostly negated. Smad3, which is upregulated by TGF-β1 and elevated during fibrogenesis (reviewed in ref. ^35^) was also increased in the presence of GzmB. GzmB-mediated decorin cleavage therefore provides a mechanistic role for GzmB in the development of fibrotic conditions in PI. Healed wounds typically display reduced tensile strength with only 80% retained after 2 years post-injury^36^. This is especially a problem with PI as they tend to redevelop in the specific regions associated with bony prominences. As such, therapeutic inhibition of GzmB, and its expected effect on improved ECM remodeling and increased tensile strength may therefore reduce PI recurrence in the elderly.

GzmB is emerging as an important mediator of skin injury, inflammation, and repair. GzmB is dramatically elevated in severe PI, and contributes to impaired wound repair, functioning through increased ECM degradation, elevated microvascular hemorrhage, and the induction of a fibrotic phenotype. Future studies investigating GzmB would likely benefit from focusing on disease in a chronic or age-related context, as the role of GzmB is apparently more pronounced than evident in acute models of injury. GzmB therefore represents a valid therapeutic target for the treatment of PI in an aging population.

## Methods

### Study approvals

Human PI tissue, control skin, and PI wound fluid were obtained from Vancouver General Hospital Burns Clinic or Vancouver Coastal Health with approval from the University of British Columbia Human Research Ethics Committee (H12-00540) and after obtaining written, informed patient consent. Animal studies were performed in accordance with the guidelines for animal experimentation approved by the Animal Experimentation Committee of the University of British Columbia (A17-0318).

### ELISA

Human GzmB (Thermo, Waltham, MA, USA) and mouse ICAM-1 (R&D Systems, Minneapolis, MN, USA) ELISAs were performed as per the kit instructions.

### Immunohistochemistry, IF, TBO and Picosirius red staining

Immunohistochemistry and IF were performed as previously reported^32^. Briefly, paraffin-embedded slides were deparaffinized and rehydrated through sequential immersion in xylene and ethanol (100%, 95%, 75% (v/v)). Antigen retrieval was performed by incubation in 10 mM sodium citrate, pH 5.0, for 10 min at 90 °C followed by 3% (v/v) H_2_O_2_ for 10 min at 20 °C. Non-specific binding was reduced by blocking with 10% (v/v) horse or goat serum for 30 min at 20 °C, depending on the animal used to generate the secondary antibodies. Tissue was incubated with primary antibodies as indicated in Supplementary Table 3 for 1 h at 20 °C. For immunohistochemistry (IHC), slides were incubated with biotinylated anti-rabbit or goat secondary antibodies (1/350 dilution) for 30 min at 20 °C before labeling with ABC-HRP peroxidase (Vector Labs, Burlingame, CA, USA) for 30 min and color reaction with NovaRed peroxidase (Vector Labs, Burlingame, CA, USA) for 5 min at 20 °C as per the kit instructions. Slides were mounted and scanned with a Leica Aperio CS2 Slide Scanner (Wetzlar, Germany). For IF, 488 nm and 594 nm fluorophore-conjugated anti-rabbit or goat secondary antibodies (1/1000 dilution) were added to slides for 1 h at 20 °C before mounting and analysis on a EVOS FL fluorescence microscope (Thermo Fisher Scientific, Waltham, MA, USA). TBO, Picosirius red^7^, Prussian blue, and Masson’s trichrome^13^ staining were performed using standard protocols. Images were captured using the Zeiss AxioObserver Z.1 laser scanning confocal microscope, with images captured using the Zen Software (Zeiss, Jena, Germany).

### Animals

ApoE−/− and GzmB−/− mice were purchased from The Jackson Laboratory (Bar Harbor, ME, USA). DKO mice were generated by breeding GzmB−/− with ApoE−/− mice at the Genetic Engineered Models facility (Centre for Heart Lung Innovation, UBC, Vancouver, BC, Canada). Male mice used in the study were fed ad libitum a regular chow diet (equal parts PicoLab Mouse Diet 20: 5058 (9% fat) and PicoLab Rodent Diet 20: 5053 (5% fat), LabDiet, Richmond, IN, USA) until 8 weeks of age, then switched to an HFD (21.2% fat and 0.2% cholesterol, TD.88137; Harlan Teklad; Madison, WI, USA) for 30 weeks, at which point they underwent the I/R procedure. All mice were on a C57BL/6 background.

### Murine model of I/R-mediated PI

Mice were anesthetized with inhaled isoflurane, and the dorsum shaved and hair removed with Nair (Church & Dwight, Ewing, NJ, USA). A template drawn with a marker was used to mark the location of the magnets on the dorsum thus assuring consistent placement on each animal. The skin was gently pulled up and placed between two identical round magnets (12 mm diameter, 5.0 mm thick, 1000 G magnetic force, 50 mm Hg compressive pressure) (Master Magnetics, Castle Rock, CO, USA) such that a 5 mm skin bridge was present between the magnets. I/R cycles consisted of a 3 h period of magnet placement followed by a rest period (30 min or overnight) (Supplementary Figure 1). Animals were not immobilized or anesthetized during I/R cycles and were allowed food and water ad libitum. Digital photographs of the wounds were captured daily. A ruler was placed adjacent to the PI to allow direct wound measurements to be recorded. Animals euthanasia was performed and wound tissue collected at d7, d14, and d21 post-initiation of I/R. Wound tissue was bisected into two equal size sections; one half was micro-dissected to remove excess skin then snap frozen in liquid nitrogen and used to prepare tissue extracts. The other half of the PI tissue was fixed in 10% (v/v) buffered formalin and mounted in paraffin.

### Laser Doppler imaging

Tissue blood perfusion was determined using the Periscan® PIM 3 Blood Perfusion Imager apparatus. Blood perfusion was displayed both as a numerical PU (volts) and also as a color-coded image where bright red indicated high perfusion and dark blue indicated low perfusion. The scanner head distance was 15 cm; laser beam power was 1 mV; wavelength was 670 nm; resolution was 0.36 mm; scanning area was 1.4 cm^2^; scanning time was 30 s.

### Skin tensiometry

PI tissue tensile breaking force was determined using a Mecmesin Motorised Force Tester (Mecmesin Corporation, Slinfold, UK). Excised PI tissue (1 × 4 cm) was loaded into a 200-N Spring Action Vice Clamp (Mecmesin Corporation, Slinfold, UK) and stretched apart at 3 cm/min with a MultiTest 2.5-d Test System Stand (Mecmesin Corporation, Slinfold, UK). The minimum force needed to initiate breakage of skin leading to reduction in force was determined with the Advanced Force Gauge 100 N (Mecmesin Corporation, Slinfold, UK) and recorded using Emperor Lite software (Mecmesin).

### Proteome profiler

Proteome profiler antibody arrays were used to detect a panel of cytokines/chemokines in mouse tissue extracts (200 µg total cell protein) as per the kit instructions (mouse cytokine array panel A, R&D Systems, Minneapolis, MN, USA). PI tissue extracts from ApoE−/− and DKO mice at d7 and d14 were tested in duplicate and presented as the mean.

### Morphometric analysis

IHC/IF intensity was calculated in the granulation tissue of PI tissue; GzmB and TBO as total positive cells per unit area within two representative rectangles of 200 × 160 mm^2^; fibronectin, VEGF, decorin, α-SMA, TGF-β1, Smad3 as staining intensity per unit area within two representative rectangles in the wound area, as above. Data were presented as the number of positive cells in the PI tissue as a percentage of positive cells in unwounded controls. Inflammatory cell infiltration determined by measure of the ratio of high-density blue (indicating nuclear) to red (indicating cytoplasmic) staining. Lesional gape was determined as the distance of one leading edge of the epithelium to the other, epidermal gape as the length of the epithelium affected by hyperproliferative epidermis, wound margin as the distance of the wound from the mid-point depth of the dermis to the other side, wound area as the total wound area below the epidermis and above the panniculus carnosus. Vascular micro-hemorrhage was determined as the total number of hemosiderin-positive regions per unit area within the whole wound tissue section. Collagen density measured as Picosirius red staining intensity per unit area within two representative rectangles in the granulation tissue of wound sections, as above. Mature collagen was determined in Masson’s trichrome-stained slides as the ratio of mature to immature collagen within two representative rectangles in the granulation tissue of wound sections, as above. Color deconvolution was used to isolate the colors for quantification using ImageJ software (NIH, USA). Each slide was blinded before analysis, with *n* ≥ 4 images per group analyzed.

### Statistical analysis

Statistical differences in all experiments were determined using either the Student’s *t*-test (two-sided, non-paired) or ANOVA, with Bonferroni post-test used for group comparison analyses and *P* < 0.05 considered significant. For data not following a normal distribution, the Mann–Whitney *U*-test was performed. Error bars represent the mean and SEM.

### Reporting summary

Further information on research design is available in the Nature Research Reporting Summary linked to this article.

## Supplementary information

Supplemental Material

reporting summary

## Data Availability

Available from the corresponding author on reasonable request
