# Supplementary material for: Granzyme B mediates impaired healing of pressure injuries in aged skin
Source: NPJ Aging Mech Dis. 2021 Mar 5;7:6. doi: 10.1038/s41514-021-00059-6 (PMC7935969; doi:10.1038/s41514-021-00059-6)
Supplement: Supplementary file 1 — Supplemental Material [file 41514_2021_59_MOESM1_ESM.pdf]

Supplementary Table 1. Human skin controls

Sample	Sex	Subject information	Age (years)
1	F	abdomen	70
2	F	-	61
3	F	-	59
4	F	-	55
5	F	abdomen	49
6	F	-	45
7	M	-	40
8	M	-	25
9	F	-	21
10	M	-	21

Supplementary Table 2. PI severity score

PI severity score	Parameter
0	Intact, healthy skin with normal capillary refill
1	Intact skin, non-blanchable erythema
2	Superficial/partial skin loss involving epidermis and dermis
3	Full thickness loss, damage and necrosis of subcutaneous tissue
4	Full thickness loss, extensive tissue necrosis, bone/muscle exposure

Supplementary Table 3. Antibodies for immunohistochemistry and immune-fluorescence.

Antibody	Species	Final Concentration	Source and Catalog #
GzmB	human	1/150	Abcam, ab4059
	mouse	1/150	Abcam, ab4059
CD68	human	1 μg/mL	Abcam, ab125212
fibronectin	mouse	1/100	Abcam, ab2413
VEGF	mouse	1/50	R&D, AF493
decorin	human	1/250	Abcam, ab67449
	mouse	10 μg/mL	R&D, AF1060
α-SMA	mouse	1/1000	Abcam, ab5694
TGF-β1	mouse	1/100	BD Biosciences, 555052
Smad3	mouse	1/100	Abcam, ab52903

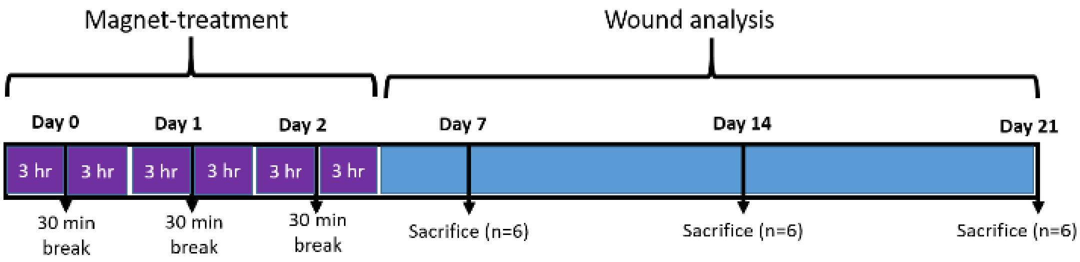

Supplementary Figure 1: Protocol used to induce PI in mice using I/R.

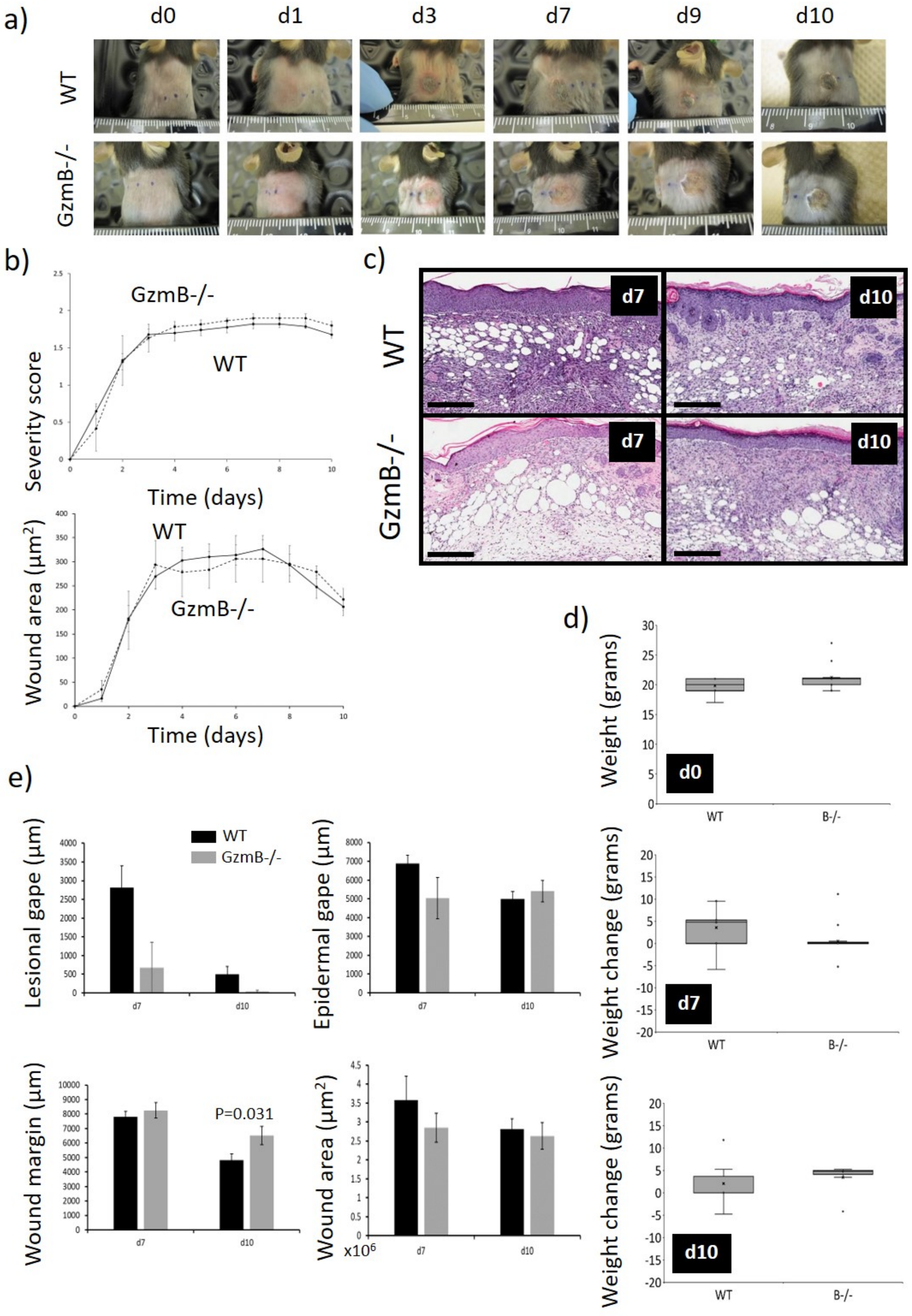
a)
d0
d1
d3
d7
d9
d10
WT
GzmB-/-
b)
GzmB-/-
WT
Severity score
Time (days)
WT
GzmB-/-
Wound area (μm²)
Time (days)
c)
d7
d10
WT
d7
d10
GzmB-/-
d)
Weight (grams)
d0
WT
B-/-
Weight change (grams)
d7
WT
B-/-
Weight change (grams)
d10
WT
B-/-
e)
WT
GzmB-/-
Lesional gape (μm)
d7
d10
Epidermal gape (μm)
d7
d10
Wound margin (μm)
P=0.031
d7
d10
Wound area (μm²)
x10⁶
d7
d10

Supplementary Figure 2: No Difference in Wound Severity between GzmB-/- and WT Mice PI. (a) PI photos. (b) PI severity score and macroscopic measure of wound area. Data presented as mean ± SEM, n=6. * $P < 0.05$, ** $P < 0.005$. (c) Representative images of H&E stained mouse PI tissue. Scale bars = 500 μm. (d) Weight of WT and GzmB-/- mice at weeks post-initiation of HFD and weight change at d7 and d10. n=6 samples per group. Presented as weight (grams) or percentage weight change, n=6. (e) Wound measurements calculated from the H&E stained PI tissue. Presented as mean ± SEM, n=6.

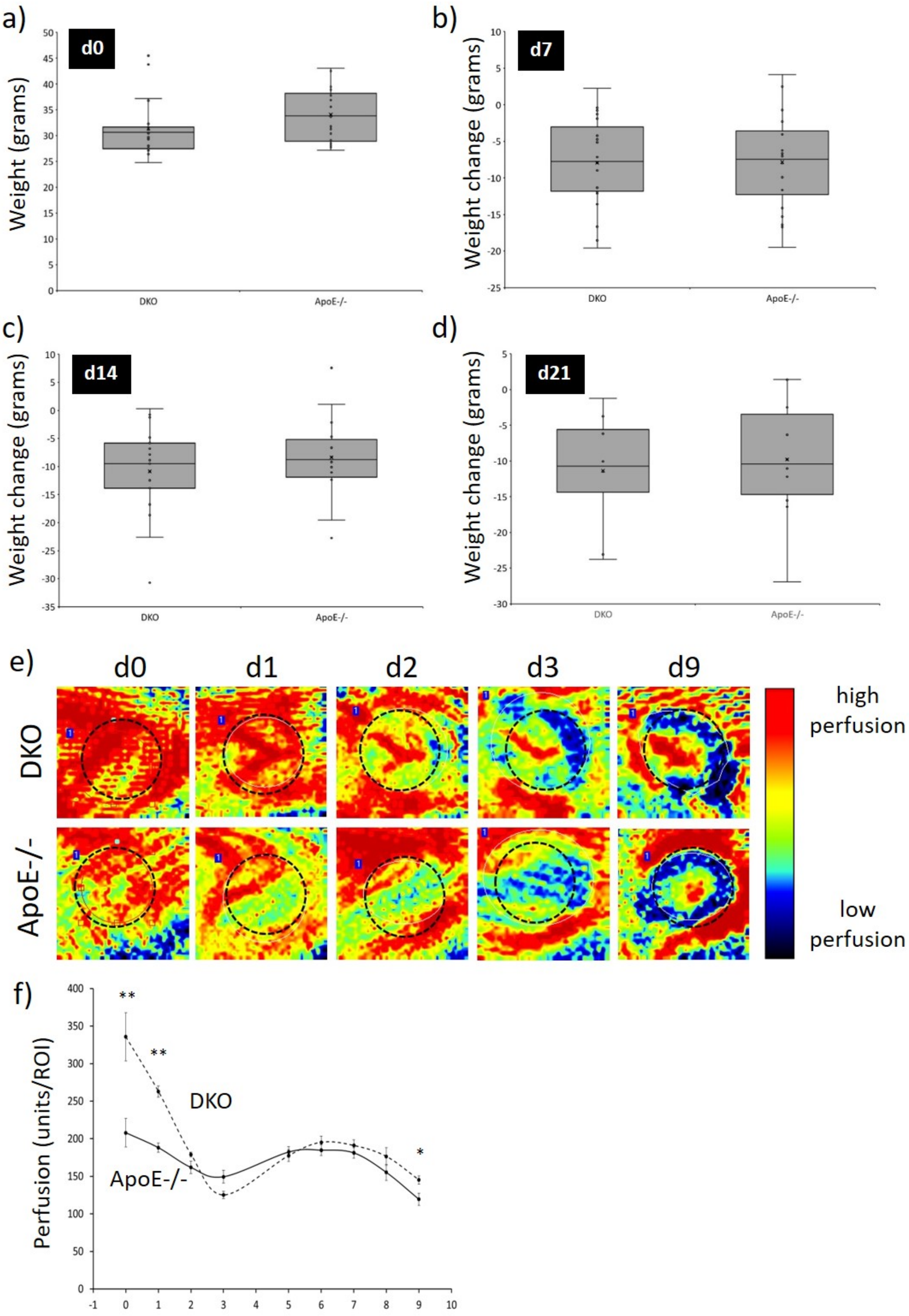

a)
d0
Weight (grams)
DKO
ApoE-/-
b)
d7
Weight change (grams)
DKO
ApoE-/-
c)
d14
Weight change (grams)
DKO
ApoE-/-
d)
d21
Weight change (grams)
DKO
ApoE-/-
e)
d0
d1
d2
d3
d9
DKO
ApoE-/-
high perfusion
low perfusion
f)
Perfusion (units/ROI)
DKO
ApoE-/-

Supplementary Figure 3: Mouse Weights and Tissue Perfusion in Response to HFD and Repeated I/R. (a) Weight of ApoE-/- and DKO mice prior to initiation of I/R. (b-d) Percentage weight change at d7, d14 and d21 post-initiation of I/R, n=6 samples per group. (e) Representative doppler images of PI tissue in ApoE-/- and DKO mice. (f) Quantification of tissue perfusion. Data presented as perfusion per unit area, mean ± SEM, n=6. *$P<0.05$, **$P<0.005$.

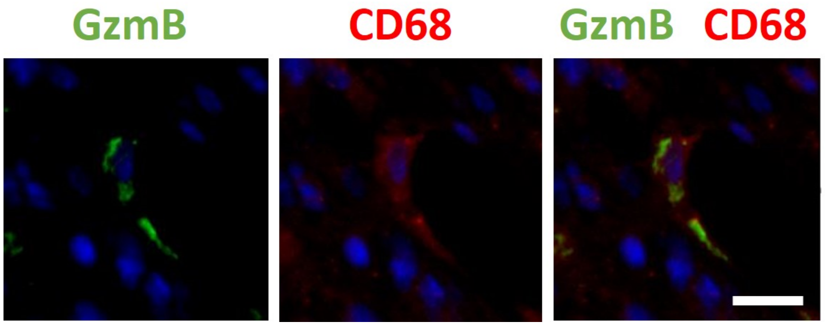

Supplementary Figure 4: GzmB Expression by CD68+ Cells in Mouse PI. GzmB (green) and CD68 (red) immune-fluorescence in mouse PI dermis at the wound margin (day 7 post-injury). Scale bar = 20 μm.

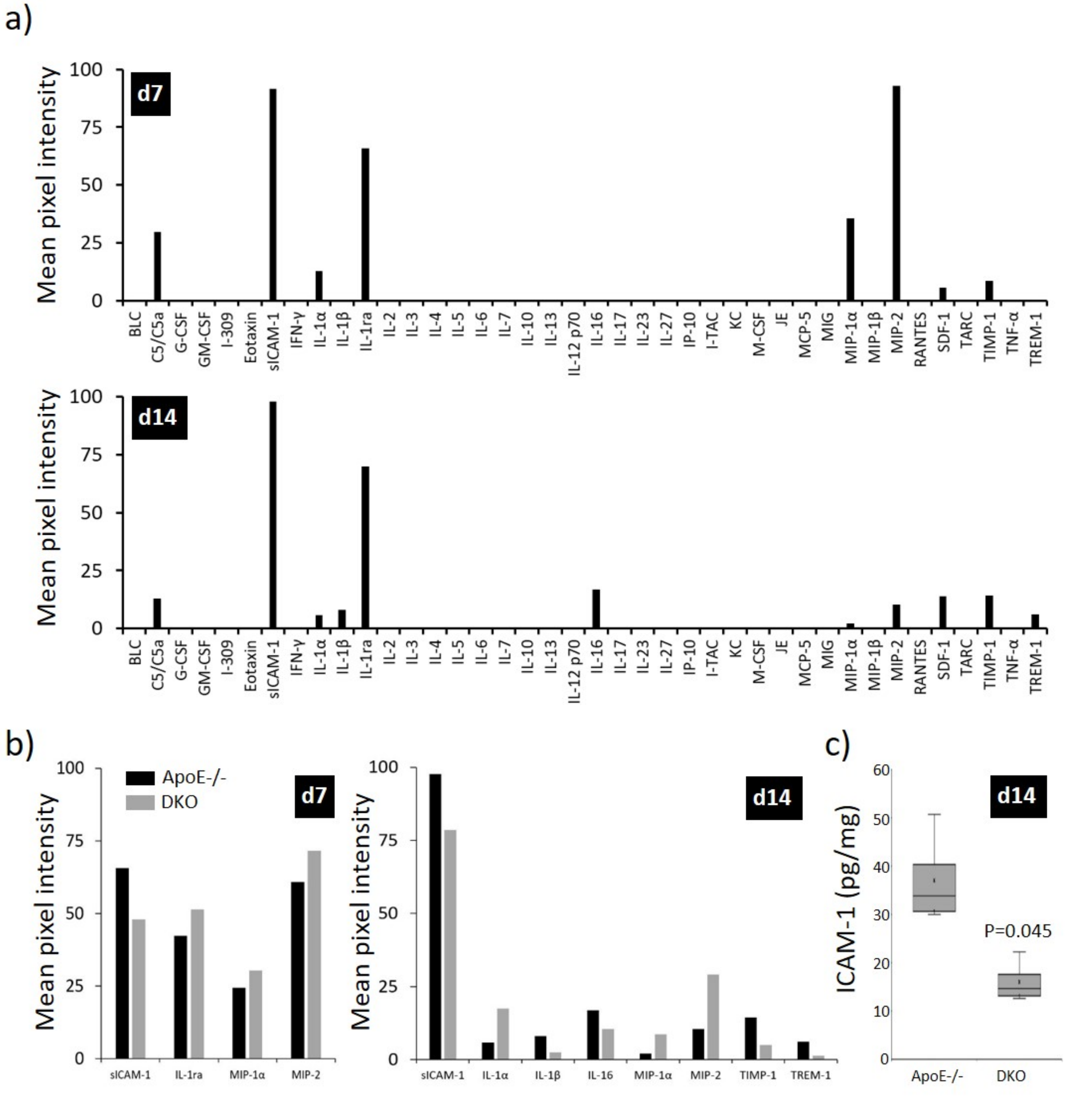
a)
d7
d14
Mean pixel intensity
100
75
50
25
0
BLC
C5/C5a
G-CSF
GM-CSF
I-309
Eotaxin
sICAM-1
IFN-γ
IL-1α
IL-1β
IL-1ra
IL-2
IL-3
IL-4
IL-5
IL-6
IL-7
IL-10
IL-13
IL-12 p70
IL-16
IL-17
IL-23
IL-27
IP-10
I-TAC
KC
M-CSF
JE
MCP-5
MIG
MIP-1α
MIP-1β
MIP-2
RANTES
SDF-1
TARC
TIMP-1
TNF-α
TREM-1
b)
ApoE-/-
DKO
d7
d14
Mean pixel intensity
sICAM-1
IL-1ra
MIP-1α
MIP-2
sICAM-1
IL-1α
IL-1β
IL-16
MIP-1α
MIP-2
TIMP-1
TREM-1
c)
d14
ICAM-1 (pg/mg)
60
50
40
30
20
10
0
P=0.045
ApoE-/-
DKO

Supplementary Figure 5: Differential Expression of Cytokines/Chemokines in DKO Mice PI. (a) Profiler screening of a panel of cytokines/chemokines at d7 and d14 post-initiation of I/R. Data displaying relative expression and expressed as mean pixel intensity, mean of n=2 per time point per group. (b) Comparison of lead candidate profiler hits, showing the relative difference in expression between ApoE-/- and DKO mice extracts at d7 and d14 post-initiation of I/R. (c) Quantification of ICAM-1 in ApoE-/- and DKO at d14 post-initiation of I/R by ELISA. Data presented as pg ICAM-1 per mg total cell protein, n=3 per group.
