# Supplementary material for: Granzyme B mediates impaired healing of pressure injuries in aged skin
Source: NPJ Aging Mech Dis. 2021 Mar 5;7:6. doi: 10.1038/s41514-021-00059-6 (PMC7935969; doi:10.1038/s41514-021-00059-6)
Supplement: Supplementary file 2 — reporting summary [file 41514_2021_59_MOESM2_ESM.pdf]

nature research

Corresponding author(s): Christopher Turner

Last updated by author(s): Dec 17, 2020

Reporting Summary

Nature Research wishes to improve the reproducibility of the work that we publish. This form provides structure for consistency and transparency in reporting. For further information on Nature Research policies, see our Editorial Policies and the Editorial Policy Checklist.

Statistics

For all statistical analyses, confirm that the following items are present in the figure legend, table legend, main text, or Methods section.

n/a	Confirmed	
☐	☒	The exact sample size (*n*) for each experimental group/condition, given as a discrete number and unit of measurement
☐	☒	A statement on whether measurements were taken from distinct samples or whether the same sample was measured repeatedly
☐	☒	The statistical test(s) used AND whether they are one- or two-sided<br>*Only common tests should be described solely by name; describe more complex techniques in the Methods section.*
☐	☒	A description of all covariates tested
☐	☒	A description of any assumptions or corrections, such as tests of normality and adjustment for multiple comparisons
☐	☒	A full description of the statistical parameters including central tendency (e.g. means) or other basic estimates (e.g. regression coefficient) AND variation (e.g. standard deviation) or associated estimates of uncertainty (e.g. confidence intervals)
☐	☒	For null hypothesis testing, the test statistic (e.g. *F*, *t*, *r*) with confidence intervals, effect sizes, degrees of freedom and *P* value noted<br>*Give P values as exact values whenever suitable.*
☐	☒	For Bayesian analysis, information on the choice of priors and Markov chain Monte Carlo settings
☐	☒	For hierarchical and complex designs, identification of the appropriate level for tests and full reporting of outcomes
☐	☒	Estimates of effect sizes (e.g. Cohen's *d*, Pearson's *r*), indicating how they were calculated

Our web collection on statistics for biologists contains articles on many of the points above.

Software and code

Policy information about availability of computer code

Data collection	Microsoft Excel
Data analysis	Microsoft Excel, IBM SPSS Statistics 27

For manuscripts utilizing custom algorithms or software that are central to the research but not yet described in published literature, software must be made available to editors and reviewers. We strongly encourage code deposition in a community repository (e.g. GitHub). See the Nature Research guidelines for submitting code & software for further information.

Data

Policy information about availability of data

All manuscripts must include a data availability statement. This statement should provide the following information, where applicable:

- Accession codes, unique identifiers, or web links for publicly available datasets
- A list of figures that have associated raw data
- A description of any restrictions on data availability

No datasets were generated during the current study

Field-specific reporting

Life sciences study design

All studies must disclose on these points even when the disclosure is negative.

Sample size	Power calculation based on 20% change in wound healing parameters
Data exclusions	No data excluded
Replication	All procedures well established in our lab were performed singularly, with relevant negative and positive controls (i.e. immunohistochemistry). All other assays performed in triplicate, with reproducible results obtained as shown by inter- and intra-assay variability calculations. Animal model extensively optimized for reproducibility prior to commencement of the study.
Randomization	Experimental groups were randomized and blinded for the the personnel performing the experiments until after experimental work and analysis was complete. Mouse provided from breeding colonies were randomly assigned to different group housed cages by animal facility staff and they were unaware of the animal genotypes.
Blinding	Personnel conducting animal surgery blinded to genotype of mice. Personnel conducting macroscopic analysis of wounds blinded to genotype of mice until the end of the trial. Personnel conducting histological analysis blinded to animal groups and time points.

Reporting for specific materials, systems and methods

We require information from authors about some types of materials, experimental systems and methods used in many studies. Here, indicate whether each material, system or method listed is relevant to your study. If you are not sure if a list item applies to your research, read the appropriate section before selecting a response.

Materials & experimental systems

n/a	Involved in the study
☐	☒ Antibodies
☒	☐ Eukaryotic cell lines
☒	☐ Palaeontology and archaeology
☐	☒ Animals and other organisms
☒	☐ Human research participants
☐	☒ Clinical data
☒	☐ Dual use research of concern

Methods

n/a	Involved in the study
☒	☐ ChIP-seq
☒	☐ Flow cytometry
☒	☐ MRI-based neuroimaging

Antibodies

Antibodies used	Detailed information in Supp Table 3.<br>GzmB: Abcam, ab4059, lot# GR256048-1<br>CD68: Abcam, ab125212, lot# GR77386<br>Fibronectin: Abcam, ab2413, lot# 208777<br>VEGF: R&D, AF493, lot# YU1311111<br>decorin (human): Abcam, ab67449, lot# GR876576<br>decorin (mouse): R&D, AF1060, lot# GBG0218011<br>a-SMA: Abcam, ab5694, lot# GR87726-1<br>TGF-B1: Clone A75-2, BD Biosciences, 555052, lot# 3280905<br>Smad3: Clone EP8234, phospho S423 + S425, Abcam, ab52903, lot# GR1288794
Validation	GzmB: validated in diabetic wounds comparing GzmB-/- and wild-type mice, https://www.abcam.com/granzyme-b-antibody-ab4059.html, reacts with mouse and human, suitable for IHC-P<br>CD68: validated in mouse spleen, https://www.abcam.com/cd68-antibody-ab125212.html, reacts with mouse and human, suitable for IHC-P<br>Fibronectin: validated in mouse kidney, https://www.abcam.com/fibronectin-antibody-ab2413.html, reacts with mouse, suitable for IHC-P<br>VEGF: validated in mouse kidney, https://www.rndsystems.com/cn/products/mouse-vegf-164-antibody_af-493-na, suitable for IHC, reacts with mouse<br>decorin (human): validated in human skin, discontinued from manufacturer, suitable for IHC, reacts with human<br>decorin (mouse): validated in mouse skin, https://www.rndsystems.com/products/mouse-decorin-antibody_af1060, reacts with mouse, suitable for IHC<br>a-SMA, validated in fibrotic scar tissue, https://www.abcam.com/alpha-smooth-muscle-actin-antibody-ab5694.html, reacts with mouse and human, suitable for IHC-P<br>TGF-B1: Clone A75-2, validated in spleen, reacts with human and mouse, https://www.citeab.com/antibodies/2412878-555052-bd-pharmingen-purified-rat-anti-mouse-human, optimized for IHC-P<br>Smad3: validated in spleen, https://www.abcam.com/smad3-phospho-s423--s425-antibody-ep823y-ab52903.html, reacts with human and mouse, suitable for IHC-P, requires heat mediated antigen retrieval with Tris/EDTA buffer pH 9.0 before commencing

April 2020

	with IHC staining protocol.

Animals and other organisms

Policy information about studies involving animals; ARRIVE guidelines recommended for reporting animal research

Laboratory animals	Mouse, all C57Bl6 background, wild-type, GzmB knockout, ApoE knockout, GzmB/ApoE double knockout, females and males included, age = 8 weeks and 38 weeks
Wild animals	Not applicable
Field-collected samples	Not applicable
Ethics oversight	Animal studies were performed in accordance with the guidelines for animal experimentation approved by the Animal Experimentation Committee of the University of British Columbia (A17-0318).

Note that full information on the approval of the study protocol must also be provided in the manuscript.

Clinical data

Policy information about clinical studies

All manuscripts should comply with the ICMJEguidelines for publication of clinical research and a completedCONSORT checklist must be included with all submissions.

Clinical trial registration	Not applicable - obtained discarded tissue from surgeries only
Study protocol	Human PI tissue, control skin and PI wound fluid was obtained with approval from the University of British Columbia Human Research Ethics Committee (H12-00540) and after obtaining written, informed patient consent.
Data collection	Human PI tissue, control skin and PI wound fluid was obtained from Vancouver General Hospital Burns Clinic or Vancouver Coastal Health from 2018-2020
Outcomes	Tissue embedded in paraffin and sectioned for IHC. Used to measure GzmB+ cells and other proteins
